# A Review of the Development of Multitarget Molecules against HIV-TB Coinfection Pathogens

**DOI:** 10.3390/molecules28083342

**Published:** 2023-04-10

**Authors:** Debora Inacio Leite, Stefany de Castro Bazan Moura, Maria da Conceição Avelino Dias, Carolina Catta Preta Costa, Gustavo Peixoto Machado, Luiz Claudio Ferreira Pimentel, Frederico Silva Castelo Branco, Rui Moreira, Monica Macedo Bastos, Nubia Boechat

**Affiliations:** 1 Laboratorio de Sintese de Farmacos (LASFAR), Fundacao Oswaldo Cruz, Instituto de Tecnologia em Farmacos (Farmanguinhos), Fiocruz, Rua Sizenando Nabuco, 100 Manguinhos, Rio de Janeiro 21041-000, Brazil; 2 Programa de Pos-Graduação em Farmacologia e Química Medicinal, Instituto de Ciências Biomédicas (ICB), Universidade Federal do Rio de Janeiro (UFRJ), Av. Carlos Chagas Filho, Rio de Janeiro 21941-902, Brazil; 3 Departamento de Química Medicinal, Faculdade de Farmácia, Universidade de Lisboa, Av. Professor Gama Pinto, 1649-003 Lisboa, Portugal

**Keywords:** AIDS, HIV, tuberculosis, coinfection, hybrid, multitarget

## Abstract

The human immunodeficiency virus (HIV) produces the pathologic basis of acquired immunodeficiency syndrome (AIDS). An increase in the viral load in the body leads to a decline in the number of T lymphocytes, compromising the patient’s immune system. Some opportunistic diseases may result, such as tuberculosis (TB), which is the most common in seropositive patients. Long-term treatment is required for HIV-TB coinfection, and cocktails of drugs for both diseases are used concomitantly. The most challenging aspects of treatment are the occurrence of drug interactions, overlapping toxicity, no adherence to treatment and cases of resistance. Recent approaches have involved using molecules that can act synergistically on two or more distinct targets. The development of multitarget molecules could overcome the disadvantages of the therapies used to treat HIV-TB coinfection. This report is the first review on using molecules with activities against HIV and *Mycobacterium tuberculosis* (MTB) for molecular hybridization and multitarget strategies. Here, we discuss the importance and development of multiple targets as a means of improving adherence to therapy in cases of the coexistence of these pathologies. In this context, several studies on the development of structural entities to treat HIV-TB simultaneously are discussed.

## 1. Introduction

### 1.1. Acquired Immunodeficiency Syndrome

Human immunodeficiency virus (HIV) belongs to the family *Retroviridae* and genus *Lentivirus* and produces the pathological basis of acquired immunodeficiency syndrome (AIDS) [1,2]. AIDS remains a worldwide health problem of unprecedented dimension. The first clinical report of the disease occurred in 1981 [3,4]. However, only after pathogen discovery did research begin to produce novel effective drugs [4]. According to the Joint United Nations Program on HIV and AIDS (UNAIDS), 38.4 million people worldwide were living with AIDS in 2021 [5] of which more than two-thirds lived on the African continent [6].

**Figure 1:**
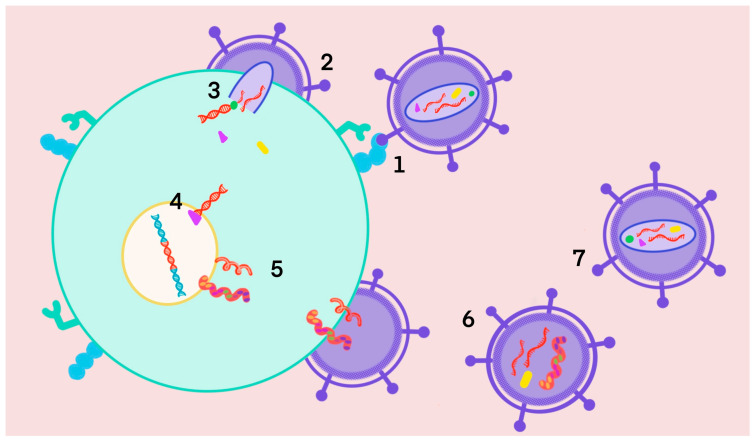
HIV replication cycle.

For a better understanding of how the treatment of AIDS works, it is important to know how the virus replication cycle occurs. The viral particle fuses with the host cell through the interaction of the gp120 envelope glycoprotein with the CD4+ cell receptor and CCR5 or CXCR4 coreceptors (Figure 1; step 1). Coreceptor binding causes a deformation in the conformation of gp120, which leads to contortion of the gp41 trimeric glycoprotein, introducing its hydrophobic endings into the cell membrane and bringing the viral particle closer to the target cell to facilitate fusion (Figure 1; step 2). The fusion process enables the introduction of viral genetic material and replication enzymes into the host cell. The first step is the synthesis of proviral DNA through RNA by the action of the enzyme reverse transcriptase (RT) (Figure 1; step 3). Once synthesized, this pro-viral DNA becomes incorporated into the host genome by the enzyme integrase (IN) (Figure 1; step 4) [7].

Activation of this cell leads to the transcription of messenger RNA (mRNA), which is transported to the cytoplasm (Figure 1; step 5). The new HIV RNA and proteins move to the surface of the cell, where a new and immature HIV is formed (Figure 1; step 6). Finally, translation of the mRNA into proteins occurs, which will undergo the action of the protease (PR) to give rise to individual functional proteins (Figure 1; step 7) [7].

Combination antiretroviral therapy (cART), usually called an AIDS cocktail, is composed of more than one drug and must be administered daily for the lifetime of the infected patient once HIV has been integrated into the host cell genome DNA. The available anti-HIV drugs target the essential HIV enzymes RT, IN and PR. However, other stages of HIV infection can be addressed, for example, entry and capsid inhibitors [8]. There is no cure for this disease through a pharmacological approach or a vaccine to help avoid HIV transmission, and drug-based therapy is the only means to control infection progression.

Approximately 1% of the general population exhibits homozygous inactivating deletions in CCR5. This CCR5 mutation inhibits HIV entry into CD4 cells and has been responsible for some cases of HIV cure due to stem cell transplantation in patients who received cells from donors possessing this variant allele. Despite this very exciting alternative cure, stem cell transplantation is not considered an option to achieve HIV remission and cure as it is an invasive, complex and risky procedure [9,10].

**Figure 2:**
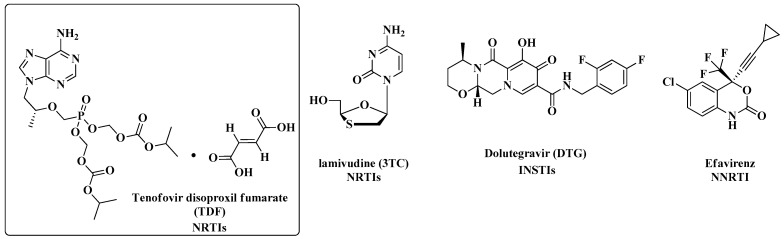
Chemical structure of the main antiretroviral drugs currently used in AIDS therapy.

Current AIDS treatment includes the use of a cocktail of drugs that act on different targets [11]. The preferred regimen is based on the combination of two nucleoside/nucleotide reverse transcriptase inhibitors (NRTIs) plus a third IN strand transfer inhibitor (INSTI). Studies show that the use of dolutegravir (DTG) as an INSTI is preferred because of its durable virologic efficacy, high barrier to resistance, and favorable tolerability and toxicity profiles. Efavirenz (EFV) at a low dose (400 mg) in combination with an NRTI backbone is recommended as the alternative first-line regimen for adults and adolescents living with HIV and initiating antiretroviral therapy (ART) [12]. Figure 2 shows the chemical structure of the main antiretroviral (ARV) drugs used in AIDS therapy [11,13].

However, finding a cure for AIDS has remained challenging thus far [14]. CD4+ lymphocyte infection and destruction result in the severe immunosuppression features of AIDS, allowing the establishment of secondary infections. The latent viral reservoir is the principal barrier to an HIV cure. Although CD4+ T cells are the primary targets of the virus, cells of myeloid origin also play a significant role in HIV infection. The virus is capable of persisting in macrophages, and this event represents a huge challenge for the development of a strategy for viral eradication. The virus commonly remains dormant in CD4+ T and myeloid cells as genome-integrated and competent for replication. HIV-infected monocytes and macrophages are significant mediators of inflammation, and these aberrant inflammatory events are key drivers of comorbidities in people living with HIV (PLWH) [15,16]. Among these comorbidities, tuberculosis (TB) stands out.

### 1.2. Tuberculosis

PLHIV are immunosuppressed and therefore susceptible to other infections. One of the most common infections is pulmonary TB, which is caused by Mycobacterium tuberculosis (MTB) [17]. TB is one of the most relevant health problems, being the 13th leading cause of death in the world, where approximately 1.5 million people (1.3 million HIV-negative individuals and 214,000 HIV-infected individuals) died from TB in 2020 [18].

Until the coronavirus pandemic (COVID-19) occurred, TB was the leading cause of death from a single infectious agent. COVID-19 has significantly impacted the supply of essential services provided to TB patients, in addition to causing a drastic global reduction in the number of newly diagnosed patients. Approximately 10 million people became ill with TB in 2020 [18].

MBT, also called Koch’s bacillus, is disseminated through the exposure of contaminated biological materials. The most common and rapid form of spread of the disease is through an individual with the pulmonary form of TB that expels bacilli by coughing and sneezing. The disease is currently treated with a standard therapy combination of four antimicrobial drugs over a six-month course that does not facilitate patient compliance [17].

**Figure 3:**
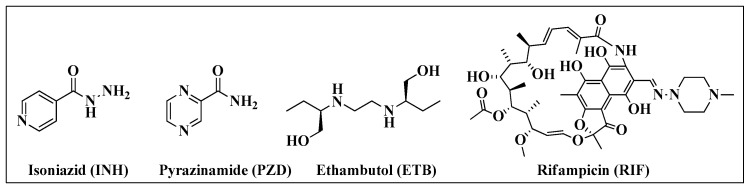
Chemical structures of anti-TB drugs.

The protocol currently used as a first-line treatment of TB consists of administering of isoniazid (INH), pyrazinamide (PZD), ethambutol (ETB) and rifampicin (RIF) (Figure 3). The protocol comprises two phases: the first is called the “intensive phase” and takes two months using a fixed-dose drug combination (FDC) of all four drugs. Over the next four months, an FDC with only RIF and INH is administered, and this phase is called the “continuation phase” [19].

Failure to adhere to treatment is a major cause of the development of resistant strains, which may be multidrug-resistant (MDR-MTB) and extensively resistant (XDR-MTB). In general, these bacterial resistances are more frequent for INH and RIF. Resistance to one anti-TB drug is described as monoresistance, and resistance to more than one is polyresistance, except for the association between INH and RIF, which is known as multiresistance [20].

In patients with confirmed RIF-susceptible, INH-resistant tuberculosis, treatment with RIF, ETB, PZD and levofloxacin is recommended for a duration of 6 months [20]. The recommendation of the World Health Organization (WHO) for the treatment of rifampicin-monoresistant (INH-susceptible) patients is that they should be treated in the same way as those that demonstrate multiresistance, but the use of INH is recommended. Some studies suggest that treatment with INH and drugs of Group A are effective. Within this scenario of the constant development of resistant strains and the high toxicity of the drugs used in therapy, the search for new therapeutic options becomes imperative. Many groups have been searching for new anti-MTB drugs [21,22,23,24].

Recently, the WHO carried out an assessment of the relative benefits and harms of the drugs available for multiresistance treatment. The outcomes led the organization to establish recommendations for each drug, and they were classified into three groups:

- Group A: Highly effective drugs that unless contraindicated, are strongly recommended for inclusion in all regimens (fluoroquinolones such as levofloxacin and moxifloxacin, bedaquiline and linezolid).
- Group B: Conditionally recommended as agents of second choice (clofazimine and cycloserine or terizidone).
- Group C: Agents that can be used when a regimen cannot be composed of Group A or B agents (for example, ETB, PZD, delamanid, amikacin and ethionamide).

### 1.3. HIV-TB Coinfection

According to the WHO, the risk of developing active TB is 26 to 31 times higher in seropositive patients than in uninfected individuals [25]. This difference is due to the decrease in the number of CD4+ T lymphocytes caused by AIDS, which prevents the effective formation of granuloma in MTB infection [26,27]. Global estimates in 2020 indicated 214,000 deaths in addition to seropositives caused by TB, a small increase from the 209,000 deaths reported in 2019, 85% of which occurred in Africa and Asia [28].

MTB is capable of infecting other organs to cause extrapulmonary TB. This form of TB mainly compromises seropositive patients who are more susceptible to the dissemination of this pathogen due to immunosuppression [28]. The low immunity caused by AIDS increases the spread of Koch’s bacillus, which accelerates HIV replication, exposing patients to a high-risk coinfection [29,30].

Some factors can explain the synergism between the two diseases [31,32,33,34]. First, some studies show that macrophage depletion delays recruitment of this cell type. Necrotic cell death is associated with an increase in MTB, showing that mycobacterial phagocytosis can restrict mycobacterial growth [35,36,37]. Thus, in HIV-TB coinfection, an increase in mycobacterial growth is observed due to the accessory protein of the virus, Nef. This protein inhibits the phagocytic process [38,39]. Another important factor is that macrophages start to appear as an activated phenotype M2, instead of M1, in people infected with HIV-1. This change can reduce the activity of the enzyme nitric oxide synthase, causing a loss in the apoptosis of macrophages infected by MTB [40].

HIV-TB coinfection requires long-term treatment involving the concomitant use of cocktails of drugs for both diseases. The most challenging aspects of treatment are the occurrence of drug interactions, overlapping toxicity, no adherence to treatment and consequent cases of resistance [41,42,43,44]. Simultaneous initiation of both therapies has not been recommended because it would increase drug interactions and cause an accumulation of adverse effects, which can generally lead to interruption of treatment [45].

ART should be started as soon as possible within two weeks of initiating TB treatment, regardless of the CD4 cell count, among people living with HIV. Table 1 presents the recommendations for the simultaneous use of ARV and tuberculostatic drugs [12,46].

Rifamycins are a class of antibacterial agents that were first isolated in 1959 and contain the drugs RIF, RFB and RFP [47]. In the first- and second-line regimens, the use of RIF is recommended in combination with EFV and raltegravir (RAL). However, to compose the third treatment line, the scheme is based on the simultaneous use of RFB (Table 1) with a PI. This recommendation is because the combined use of these drugs has a high potential to induce cytochrome P450 (CYP) 3A4, which is responsible for the metabolism of ARV [48]. RFB shows a lower induction power of this metabolic pathway and is less active than RIF. The plasma concentration levels of RFB are increased when it is combined with PIs [49]. Therefore, the use of RFB is advised by protocols when there is a need for the introduction of a PI in coinfection therapy.

It has been described that the use of a combination of a PI with RIF is possible if the dose of ritonavir or LPV is adjusted to repair the effects caused by the antibacterial [50]. However, this strategy is only used in cases where there is no possibility of an alternative drug combination or in cases of nonavailability of RFB. The replacement of EFV by DTG in combination with RIF in the treatment of HIV-TB coinfection is not applicable in all protocols of the world, but studies show that the use of 50 mg of DTG twice daily is well tolerated with EFV-like virological efficacy in HIV-TB-coinfected adults with concomitant use of RIF. In addition, this protocol is already recommended by the WHO and has been applied in the United States and Europe [51,52,53].

One alternative to improve adherence to HIV-TB therapy is to use a combination of drugs in an FDC. However, there is still no FDC available to treat HIV-TB coinfection. This situation may result from the complexity of developing a formulation containing more than one active pharmaceutical ingredient (API). In general, it is difficult to determine the correct dose of individual drugs, and problems are encountered with dissolution, drug interactions, etc. [54,55,56,57,58]. Another alternative to improve adherence to HIV-TB therapy is to use multitarget molecules. This treatment has emerged as a novel approach in medicinal chemistry and is discussed below.

### 1.4. Multitargets

A single molecule that can interact with two or more distinct targets is defined as a multitarget. Multitargets are a novel concept in drug development that may provide a modern approach to the treatment of multifactorial diseases or diseases for which treatment is based on drug cocktails [59,60,61,62]. Such compounds have synergistic interactions with different bioreceptors and could thus be considered safer and more effective than conventional drugs [63,64].

The paradigm of a highly specific treatment, that is, a target and a drug, has persisted for many years in the discovery of novel bioactive compounds. There is a belief that this type of treatment would be better tolerated and minimize the side effects caused by the interaction of the drug with other macromolecules. However, as multiple factors cause most diseases, it is necessary to modulate several targets concomitantly. Among the advantages that multitargets can offer concerning FCD, notable pros are more predictable pharmacokinetics, a lower probability of drug-drug interactions during the development of the formulation, and higher patient tolerance to treatment [65,66]. Regarding multitarget drugs, in 2008, Bolognesi and colleagues, based on the potential of polypharmacology in the treatment of neurodegenerative diseases, proposed the term multitarget-directed ligands (MTDLs) to refer to this class of compounds [67,68].

The molecular starting point for a multitarget project is generated using one of two distinct approaches, rational design by a combination of pharmacophores (hybridization) or screening of compound libraries of known drugs [69].

The structural design of a multitarget molecule is carried out using a broad theoretical base of medicinal chemistry concepts. Molecular hybridization is one of the main tools used in the development of bioactive compounds for treating multifactorial diseases and other diseases for which therapy consists of the administration of cocktails [70]. Hybrid molecules can be classified based on how their chemical structure relates to their targets.

**Figure 4:**
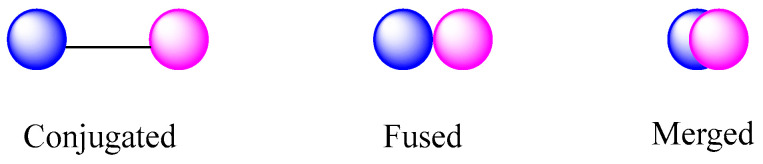
Classification of hybrids based their structure according to Morphy and Rankovic, adapted from [71].

Morphy and Rankovic have structurally classified hybrids as conjugates, fused and merged [71]. Morphy describes conjugated hybrids as molecules that can be produced by the union of two pharmacophoric groups connected by a spacer. The fused structure resembles conjugates, differing only in the joining of the two active units by a covalent bond. Finally, the merged unit is a new compound that brings together active fragments of the molecules of origin with a simpler chemical structure than those of the precursor compounds (Figure 4).

In 2018, de Castro et al. [59] classified hybrid drugs according to the mechanism of action. Thus, a subclassification emerged for conjugated hybrids according to whether a drug is cleavable or noncleavable. Two distinct drugs that are linked by a labile covalent bond, where each drug acts as a mutual carrier for the other, are classified as cleavable conjugated hybrids. These hybrids use the strategy of pro-drugs, drugs that only generate pharmacological effects after metabolic activation. In the cell interior, these hybrids undergo chemical or enzymatic hydrolysis to release two drugs that act independently on the same target or on two different targets. Noncleavable conjugated hybrids are chemical entities with two different pharmacophoric groups of two distinct drugs that do not undergo the cleavage process within the cell once they are connected by a covalent bond. Thus, the original compounds are not produced, requiring the molecular target to be considerably close to the corresponding pharmacophore to facilitate interaction with the hybrid. However, the same enzyme or molecular target is not required for hybrid action.

Conjugates with noncleavable linkers are generally more stable and are often designed to stay intact for a prolonged period. Among the advantages of choosing this type of bond are an improvement in efficacy and a decrease in the off-target effect (bioactive moiety achieves the target site intact without premature loss) [72].

A third classification can be adopted for hybrid compounds based on the location of oriented interactions. The locations can be (i) pockets of only one protein that are close together in space; (ii) pockets of different proteins, which, however, can recognize analogous endogenous ligands; or (iii) pockets of different proteins that recognize different binders [59].

In our opinion, as it is quite complex to predict the metabolism of these hybrids, it may be more reasonable to define hybrids simply based on molecular structure. Thus, molecular pharmacophoric groups are preserved for conjugated hybrids but not for fused hybrids, regardless of the metabolization process. Another important consideration is that the information about the structure–activity relationship (SAR) of the original prototypes is not always transferred directly to the planned multitarget [69].

The main objective in relation to the activity profile of a multitarget molecule is to balance the activities of the novel planned compound to ensure that each target is modulated to a similar degree in vivo at the plasma or brain levels. In general, the objective is to obtain in vitro activities with the same order of magnitude. Similar activity levels in different targets can lead to biological responses at adequate doses in the receptors in vivo. Therefore, multitargets with large affinity differences in vitro will only be active in vivo at very high doses [71].

It is important to highlight the need for the development of cellular models coinfected with both the pathogens HIV and MTB for an effective evaluation of the multiple activities of compounds planned for this purpose. Among the few reports on this subject, one was based on an in vitro model in which primary human peripheral blood mononuclear cells or monocyte-derived macrophages were infected with the *M. bovis* bacillus Calmette–Guérin (BCG) vaccine strain and HIV-1 [73]. In another study, Fan and collaborators used human macrophages coinfected with MTB and HIV-1. The in vitro coinfection system consisted of primary cells of macrophages arising from monocytes infected with HIV JR-CSF (a variant of HIV Type 1) and BCG [74]. The most commonly used strategy is to test these compounds independently, usually in cultures of mycobacteria and cells infected with HIV-1.

These independent experiments do not take into account polymicrobial interactions, including cell death and accelerated growth of pathogens, as well as additive or synergistic biological effects produced by combining compounds. These factors may explain the difficulty in advancing to clinical trials of compounds planned as multitarget HIV-TB.

The studies found in the literature (except for that described by Fan et al. [74]) aimed at the development of multitarget compounds against HIV-TB coinfection were all tested in independent trials and are discussed below.

The development of multitarget molecules would provide an alternative without the disadvantages of the therapies used to treat HIV-TB coinfection. Some studies on the development of structural hybrids to treat HIV-TB are discussed in this context. The aim of this review is to show the importance of the development of multitarget molecules as an alternative to improve adherence to therapy in cases of coexistence of these pathologies.

## 2. Multitarget HIV-TB

**Figure 5:**
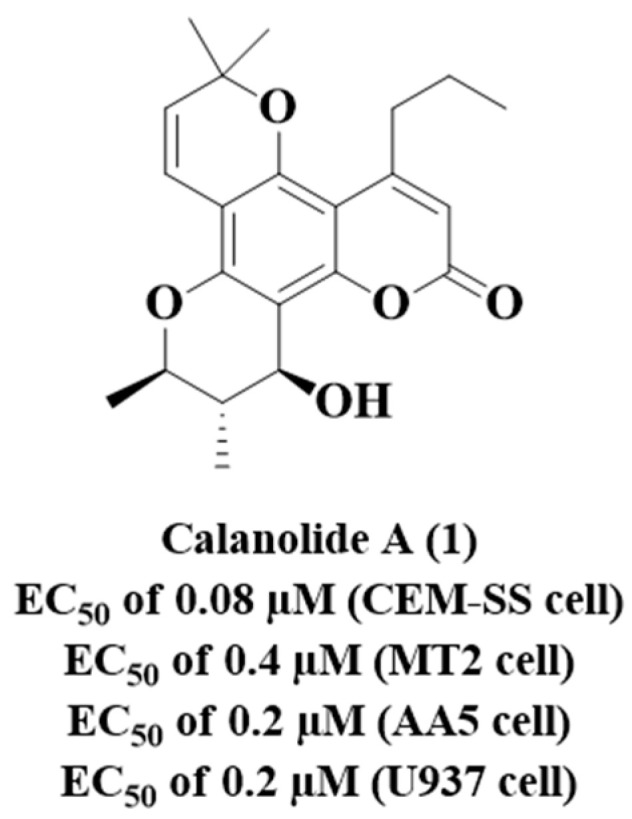
Chemical structure of Canolide A (**1**) and its anti-HIV-1 profile.

Coumarins are nuclei that have been proposed as prototypes for the inhibition of HIV [75,76] and MTB [77]. Evaluation of the activity of calanolide A (**1**) (Figure 5) against RT and nonnucleoside reverse transcriptase inhibitor (NNRTI)-resistant viruses has suggested that this compound represents a new class of HIV-1-specific inhibitors [76]. Compound **1** was determined to be active against HIV-1 (HIV-1_IIIB_) in a variety of T cell, MT2, B cell and monocytic cell lines [76] with half maximal effective concentration activities (EC_50_) against HIV-1 ranging from 0.08 to 0.4 µM (Figure 5) [76]. None of the compounds was found to have activity against HIV-2 or simian immunodeficiency virus (SIV).

Mechanistic assays showed that the compound inhibited RT with no activity in the following steps of viral replication: attachment, integration, maturation and cell-cell fusion [76,78].

**Figure 6:**
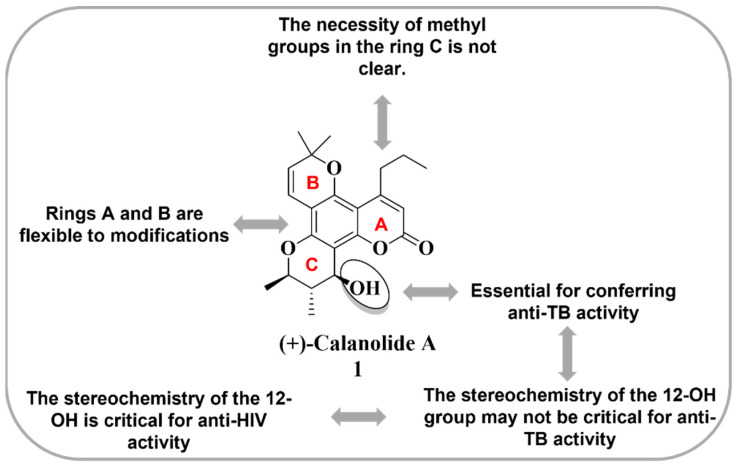
Structural requirements for pyranocoumarins to exert anti-TB and anti-HIV activity [77].

Based on excellent results for the inhibition of HIV, Xu and coworkers [77] evaluated the antimycobacterial activity of this natural product as a measure of the multitarget potential. Compound **1** (Table 2) showed 96% inhibition of mycobacteria in H_37_Rv cells at a concentration of 33.7 μM (12.5 μg/mL) with a minimal inhibitory concentration (MIC) = 8 μM (defined as the lowest concentration inhibiting >99% of the bacterial population present at the beginning of the assay). To explore the anti-MTB activity of Compound **1**, Xu et al. performed an alamar blue microdilution assay against four strains susceptible to anti-MTB drugs and strains resistant to INH, ETB, RIF and streptomycin (SMC). The results showed that the derivative (**1**) was active against the drug-sensitive strains employed in the assay as standards, as well as against MTB-resistant strains [77] (Table 2). The potential of **1** in inhibiting both pathogens (HIV and MTB) make it a good candidate for developing multitarget compounds for HIV-TB coinfection. Figure 6 describes some features regarding the structural requirements for pyranocoumarins to exert anti-TB and anti-HIV activity. Preliminary mechanistic studies have implied that calanolide A acts in RNA synthesis, similar to RIF [77].

**Figure 7:**
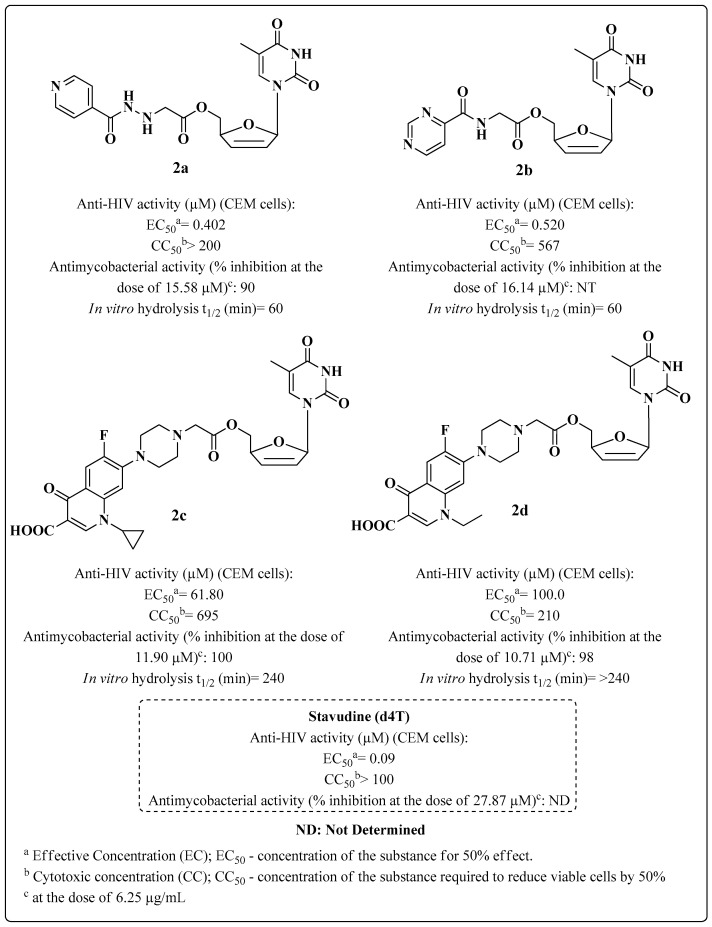
Anti-HIV and anti-MTB activity of d4T-derived Compounds **2a**–**d**, synthesized by Sriram et al. in 2004 [79].

Stavudine (d4T) is an NRTI used in anti-HIV therapy that has also been associated with anti-MTB drugs in the development of novel compounds to treat HIV-TB coinfections (**2a**–**d**) (Figure 7) through the esterification of its 5′-hydroxyl group. This approach is commonly used to enhance brain uptake and in vivo efficacy of anti-HIV nucleoside derivatives [79].

The prodrugs of d4T had their half-lives (t_1/2_) of hydrolysis determined in human plasma, and the results showed that the esters were susceptible to the action of plasma esterases with t_1/2_ in the range of 20 to 240 min (Figure 7) [79].

Compound **2a** contains an INH moiety associated with d4T and inhibited MTB by 90% and showed good inhibition of both pathogens, with an EC_50_ value of 0.4 μM against HIV-1 and lower cytotoxicity than the d4T standard. Compounds **2c**–**d** containing the moieties of fluoroquinolones and INH were evaluated as anti-MTB agents, showing a percent inhibition of MTB of above 90%. Data for INH and fluoroquinolones as standards are not shown (Figure 7). Data about the SAR and the mechanism of action were not discussed by the authors.

**Figure 8:**
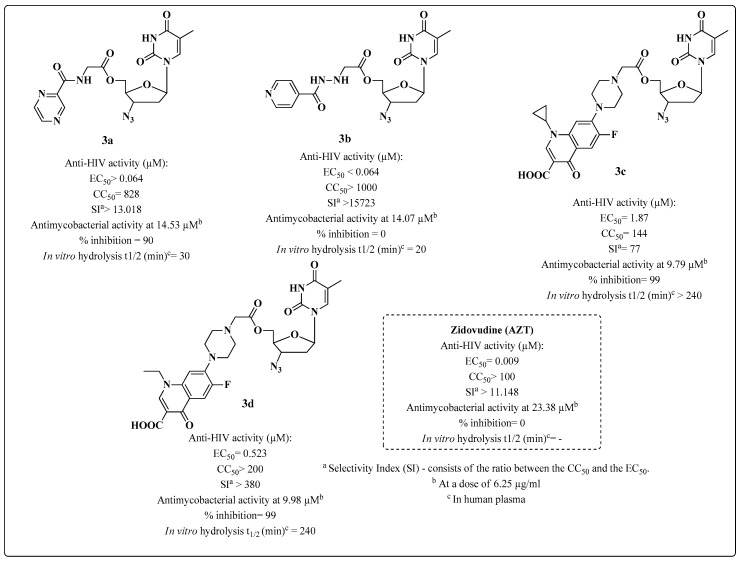
Biological activity (anti-HIV cells: MSC/anti-MTB cells: H_37_Rv) and stability of AZT derivatives synthesized by Sriram et al. in 2005 [80].

In 2005, Sriram’s group [80] investigated molecules derived from zidovudine (AZT) through the esterification of the hydroxyl at C-5′, along with the drugs ciprofloxacin (CIP), norfloxacin (NOR), INH and acetic acid from PZD [80]. The compounds were tested against HIV-1 and MTB and their plasma hydrolysis half-life times were measured (Figure 8). Compound **3d** was identified as a good candidate for a multitarget drug because of exhibiting good inhibition against both pathogens, with an EC_50_ of 0.523 μM against HIV-1 in mesenchymal stromal cells (MSC) and a selectivity index (SI) > 380, making **3d** safer to use than standard AZT. Furthermore, the compound inhibited MTB growth by 99% (Figure 8). Data about the SAR and the mechanism of action were not discussed by the authors.

**Figure 9:**
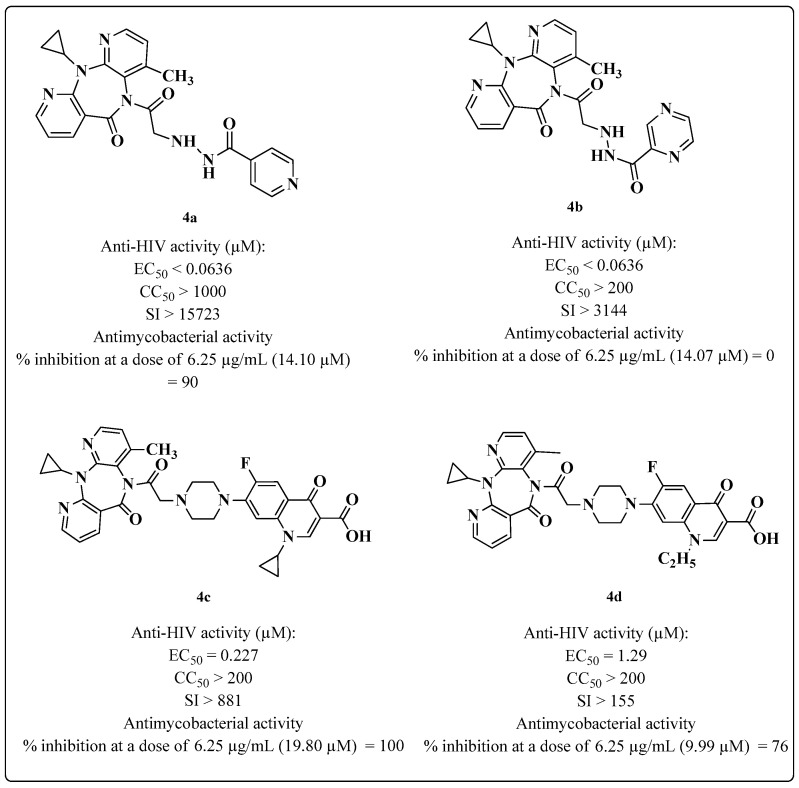
Anti-HIV (in CEM cells) and anti-MTB (in H37Rv cells) activity of the compounds synthesized by Sriram et al. in 2005 [81].

In another study, Sriram and coworkers [81] synthesized a series of hybrids (Figure 9) by combining nevirapine (NVP) with INH, PZD and derivatives of fluoroquinolones. A hybrid of NVP and INH (**4a**) stood out among the synthesized compounds. This hybrid exhibited an EC_50_ < 0.0636 μM against HIV-1 and a high SI > 15723. Additionally, **4a** presented excellent percent inhibition of MTB (90%) (Figure 9). In anti-MTB assays, the compounds with the best results were also tested against an additional 24 bacteria and exhibited lower inhibition values than the standard drugs NOR and CIP. Data about the SAR and the mechanism of action were not discussed by the authors.

**Figure 10:**
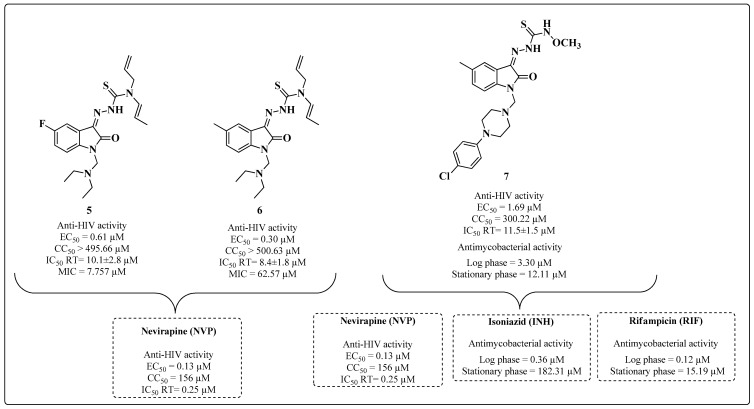
Anti-HIV and anti-MTB activities of isatin-thiosemicarbazone derivatives **5**–**7** [82,83,84,85,86,87,88].

Isatin is an important heterocycle used in the development of novel hybrids with activity against HIV-1 and MTB. Some isatin derivatives synthesized for this purpose have a Schiff base in their chemical structure. The first reported compounds were thiosemicarbazones formed from the C-3 isatin carbon [82,83,84,85,86,87,88], and the best results were obtained using Compounds **5**–**7** (Figure 10). Compound **5** exhibited an inhibitory profile of HIV-1 replication with an EC_50_ value of 0.61 μM (MT-4 cells) but was less potent than the standard NVP (EC_50_ = 0.13 μM). Nevertheless, **5** was three-fold less cytotoxic (CC_50_ > 495.66 μM) than NVP (CC_50_ of 156 μM) and had an MIC value of 7.75 μM (strain H37Ra) against MTB. Compound **6** exhibited a good EC_50_ value (0.3 μM) for the inhibition of HIV-1 with a CC_50_ > 500.63 μM but had a lower MIC (62.57 μM) than that of **5** (Figure 10).

Evaluation of Compounds **5** and **6** on the HIV-1 RT enzyme resulted in mean inhibitory concentration (IC_50_) values of 10.1 ± 2.8 μM and 8.4 ± 1.8 μM, respectively (Figure 10). Derivative **7** exhibited an EC_50_ = 1.69 μM (MT-4 cells), was two-fold less cytotoxic (CC_50_ = 300.22 μM) than the NVP standard (CC_50_ = 156 μM) and exhibited an IC_50_ = 11.5 ± 1.5 μM in an HIV-1 RT enzymatic assay. A MIC of 3.30 μM was evaluated for use against MTB during the log phase of mycobacterial growth (Strain H_37_Ra). In chronic TB, the bacillus appears in diverse metabolic phases (from active cell growth to the stationary phase). During the stationary phase, the derivative was more potent than the INH and RIF standards [88] (Figure 10). The authors did not report the activity values of the drugs used as standards in the anti-MTB trials. Data about the SAR and the mechanism of action were not discussed by the authors.

**Figure 11:**
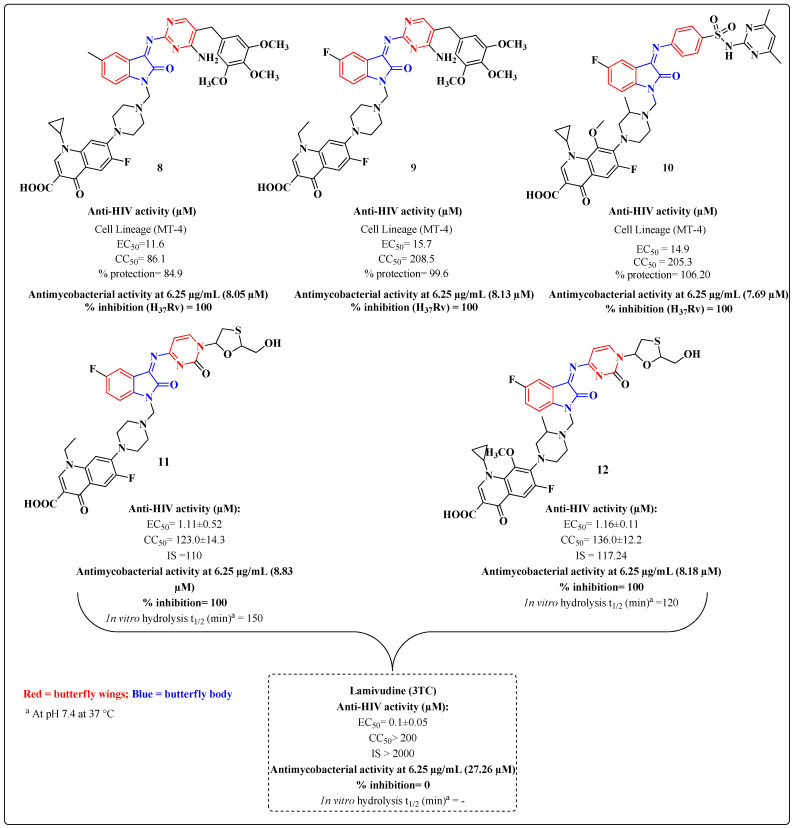
Basic skeleton of the novel isatin derivatives **8**–**12** and their anti-HIV and anti-MTB activities [83,84,86,89].

Based on the good results obtained for the isatin derivatives, novel hybrids containing this nucleus were designed and evaluated [83,84,86,89]. In these novel compounds, critical structural features of NNRTI, such as a butterfly-like structure, were preserved to evaluate the antiretroviral activity (Figure 11). Among the compounds analyzed, the derivatives **8**–**12** were evaluated for HIV inhibition in the MT-4 cell line and exhibited EC_50_ values of 11.6 μM, 15.7 μM, 14.9 μM, 1.11 μM and 1.16 μM, respectively. The best results were obtained for hybrids of 3TC with fluoroquinolones (**11** and **12**). Not all derivatives were cytotoxic at a concentration of 6.25 μg/mL in Vero cells, and an evaluation of the anti-MTB activity showed that the novel derivatives **8**–**12** inhibited MTB by 100%. In the data for the derivatives **8**–**12**, the authors did not report the activity values for the standard drugs in the anti-MTB trials or for fluoroquinolones [80,81,83]. However, 3TC was tested as an ARV standard drug (Figure 11) [89].

**Figure 12:**
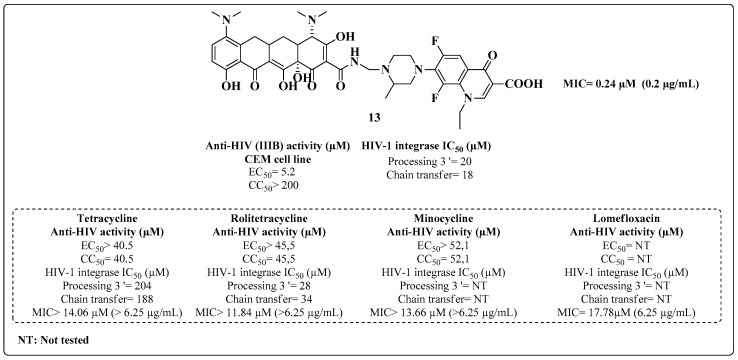
Results of anti-HIV activity (CEM cells), inhibition of the HIV-1 integrase enzyme and anti-MTB activity of Compound **13**, derived from tetracycline and fluoroquinolone [91].

Commercially available tetracycline and its derivatives [90], such as oxytetracycline, doxycycline and metacycline, have inhibitory activity against the enzyme HIV-1 integrase. Thus, many novel tetracycline derivatives containing fluoroquinolones were synthesized and tested against HIV-1 and MTB [91]. Compound **13** was the most potent of these derivatives in inhibiting virus replication, with an EC_50_ = 5.2 μM. This substance also showed inhibitory activity against HIV-1 integrase in the 3’ processing and chain-transfer stages with IC_50_ values of 20 and 18 μM, respectively (Figure 12). However, the authors did not report the activity values of the drugs used as standards in the ARV assay. The inhibitory activity of MTB was also promising, where the MIC = 0.24 μM indicated a higher efficiency than the evaluated standards (Figure 12).

**Figure 13:**
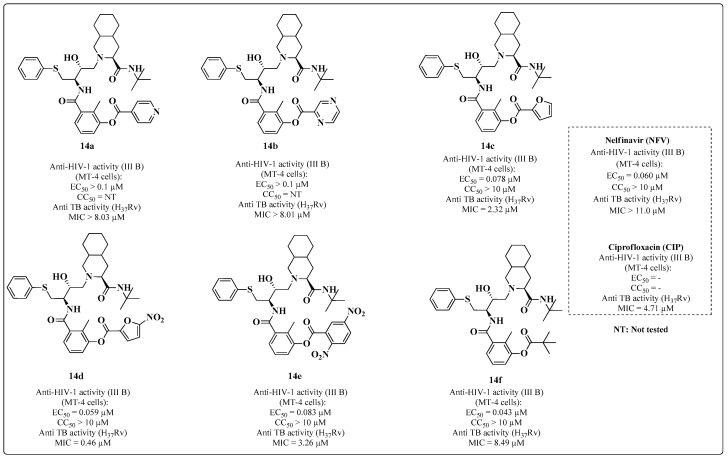
Anti-HIV and anti-MTB activities of NFV-derived compounds [92].

In the search for novel substances with dual activity, some studies have been performed using nelfinavir (NFV) as a prototype [92]. The esterified NFV derivatives **14a**–**f** were synthesized and evaluated in MT-4 cells for anti-MTB activity. Figure 13 shows the measured antiretroviral activity, where Compound **14f** was 1.4-fold more active than NFV against viral replication. The authors attributed the good biological evaluation results to the increased permeability of this compound into the cell compared to the standard. Compound **14d** was more potent than NFV in inhibiting MTB (MIC = 0.46 μM). By comparison, **14f** (MIC = 8.49 μM) was 10-fold more potent than CIP (Figure 13).

**Figure 14:**
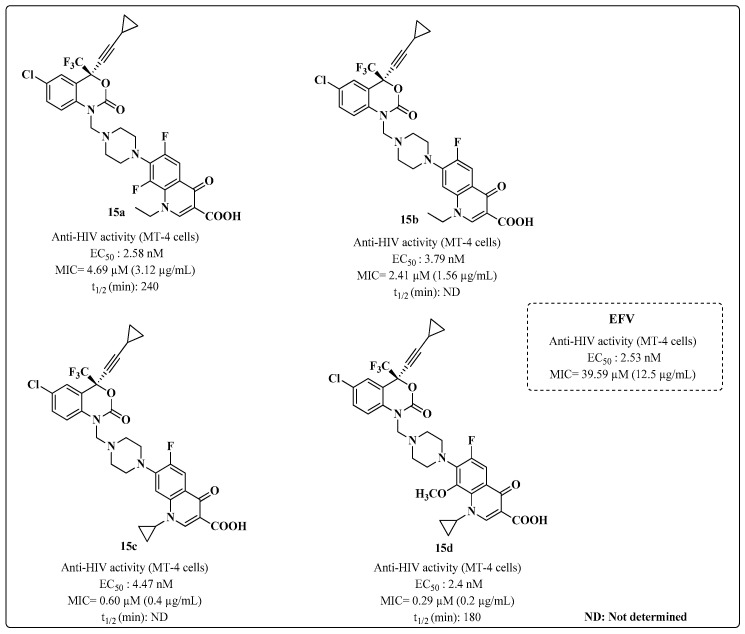
Anti-HIV and anti-MTB activities of EFV derivatives **15a**–**d** [93].

In 2009, Sriram and coworkers [93] developed the EFV derivatives **15a**–**d** by modifying the N-1 nitrogen of the drug with a Mannich base through the introduction of fluoroquinolones (Figure 14). One of the compounds (**15d**) was very promising for inhibiting HIV-1 replication, with an EC_50_ of 2.4 nM and an MIC of 0.29 μM against MTB (Figure 14). However, the authors did not report the MIC values of the drugs used as standards for MTB. The t_1/2_ of hydrolysis of the prodrugs were determined at pH 7.4, 37 °C. The data indicated that the various prodrugs of EFV were susceptible to hydrolysis with t_1/2_ in the range of 120–240 min through deaminomethylation to release efavirenz.

**Figure 15:**
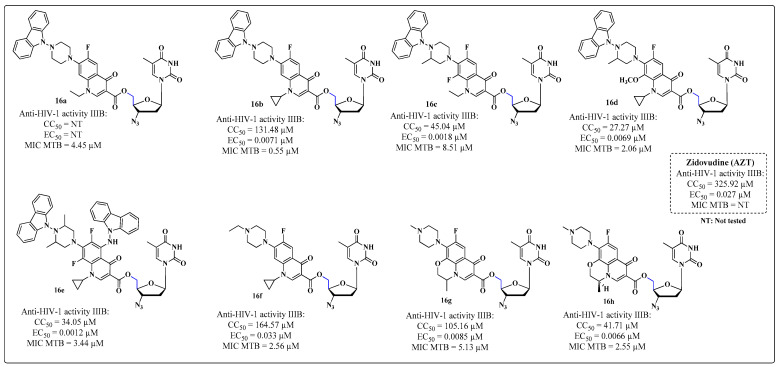
Biological activities (anti-HIV cells: MT-4/anti-MTB cells: H_37_Rv) of the derivatives **16a**–**h** synthesized by Senthilkumar and collaborators in 2009 [94].

**Figure 16:**
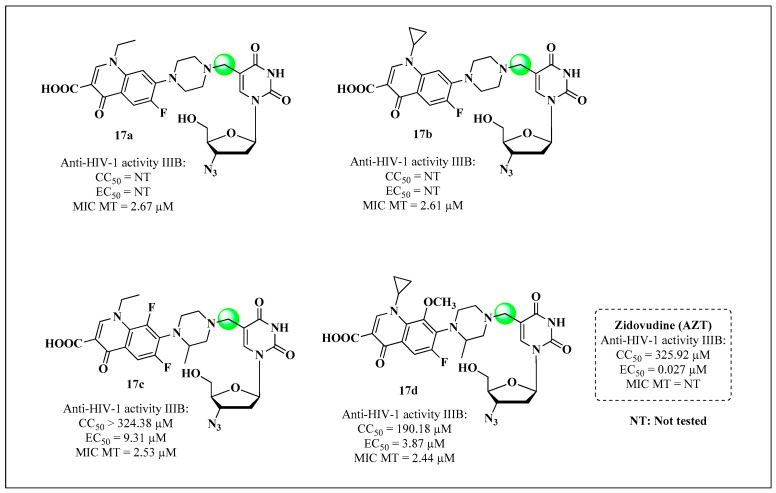
Compounds **17a**–**d** and their anti-HIV and anti-MTB activities reported by Senthilkumar and collaborators in 2009 [94].

The encouraging results of the study by Sriram and coworkers in 2005 [80] led other researchers to plan and synthesize novel derivatives for the same purpose. Senthilkumar and collaborators [94] synthesized two series of compounds. In the first series, AZT was coupled through the hydroxyl on the C-5′ carbon (blue) to fluoroquinolones, such as NOR, CIP, lomefloxacin, gatifloxacin, sparfloxacin, enrofloxacin, levofloxacin and ofloxacin, forming the esters **16a**–**h** (Figure 15). In the second series, the methyl group on the C-5 (green) carbon of the thymidine nucleus was changed by coupling NOR, CIP, lomefloxacin and gatifloxacin, giving **17a**–**d** (Figure 16) [94].

All compounds were evaluated for inhibitory activity toward HIV-1 replication, cytotoxicity in established lines of CD4+ T lymphocytes (MT-4 cells), and anti-MTB activity. Compounds **16b** and **16e** exhibited the most promising results for the inhibition of both pathogens, with EC_50_ values of 0.0071 and 0.0012 μM against HIV-1 IIIB, respectively, and MIC values of 0.55 and 3.44 μM, respectively. Both compounds were more potent than the AZT standard, and the molecule **16b** was four-fold more potent than ofloxacin [94].

With respect to the SAR, the products obtained by structural modifications of the C-5 methyl in the thymidine nucleus (**17a**–**d**) had low potency because of the inability to bind to the active site of HIV-1 RT (Figure 16). The series coupled through the hydroxyl on the C-5′ carbon are easily hydrolyzed by esterases to AZT, which becomes tri-phosphorylated and acts as an inhibitor of RT. The inhibition values of the drugs used as standards in the MTB assessment were not reported.

**Figure 17:**
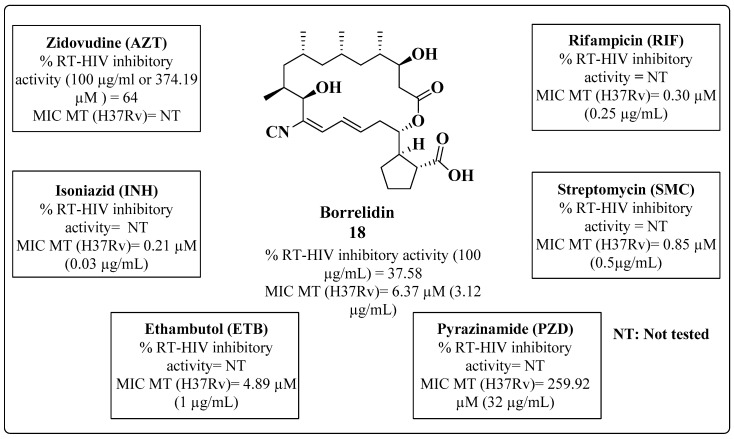
Anti-HIV and anti-MTB activities of borrelidin reported by Bhikshapathi et al. in 2010 [95].

A drug repositioning strategy was also employed in the search for a multitarget compound of HIV-1 RT with anti-MTB inhibition. The antibiotic borrelidin (**18**) was the drug of choice for these initial trials [95]. In evaluations of the drug-sensitive strains RIF, INH, PZD, ETB and SMC, **18** was found to exhibit an MIC of 6.37 μM and to be 10-fold more active than the drug PZD (Figure 17). Regarding anti-HIV-1 activity, **18** showed moderate enzymatic inhibition of RT, as shown in Figure 17. Data about the SAR and the mechanism of action were not discussed by the authors.

**Figure 18:**
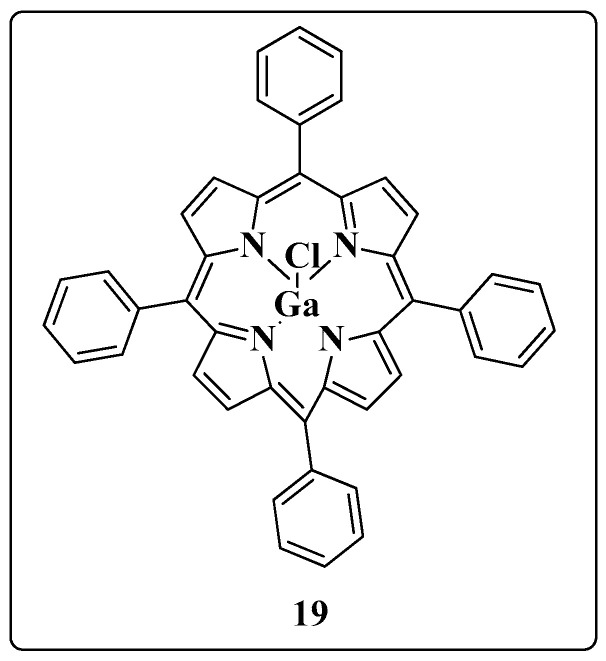
Chemical structure of the gallium nanoparticle (Ga-NP) **19** (tetraphenyl porphyrin of Ga).

To search for multitarget molecules against HIV-1 and MTB, Narayanasamy and coworkers developed a long-lived nanoparticle of gallium (Ga) (**19**) in 2015 [96] (Figure 18). This metal resembles iron (Fe) and has been reported to be potentially inhibitory to the growth and metabolism of some microorganisms, such as HIV-1 and MTB. The results showed that most Fe-dependent proteins cannot differentiate Fe from Ga, causing the death of the pathogen. In addition, Ga bound at the enzymatic site can inactivate the enzyme, unlike Fe.

Thus, Narayanasamy’s group showed that Ga-based complexes were effective against mycobacterial infections [97,98,99]. Sustained-release nanoparticles in macrophages were also evaluated for their ability to inhibit HIV-1 replication. The cell line U937 was used: monocytes derived from macrophages (MDM) were loaded for 8 h with the nanoparticles (25 mg/million cells) and infected with the virus. The results showed complete inhibition of viral replication on the first and fifth days after nanoparticle loading. Subsequently, the nanoparticles were evaluated in MDMs coinfected with HIV-1 and Mycobacterium smegmatis. The results showed significant inhibition against both pathogens throughout 15 days after a single loading of Ga nanoparticles.

**Figure 19:**
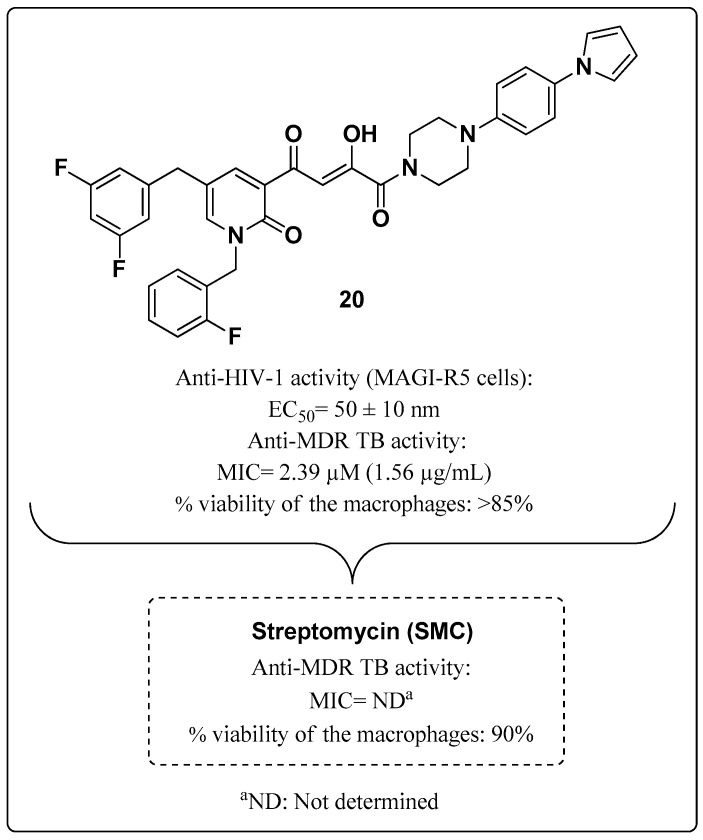
Potential multitarget HIV-TB **20** reported by Vasu Nair et al. in 2015 [101].

In 2015, Vasu Nair (one of the researchers that performed studies leading to the discovery of integrase as a target for antiretroviral chemotherapy [100]) and colleagues studied compounds with activity against MDR-MTB. Vasu Nair et al. reported the molecule **20** (Figure 19), with dual activity against HIV and MTB, for the first time. Compound **20** exhibited in vitro activity against MDR-TB (MIC 2.39 μM) and HIV-1 in MAGI-R5 cells with an EC_50_ of 50 ± 10 nM, suggesting potential dual therapeutic applications of this compound. Streptomycin was used as a standard anti-MTB drug, but its MIC was not reported. The in vitro cytotoxicity of Compound **20** was evaluated in uninfected macrophages, and a relatively low percentage of macrophage viability inhibition was found (>85%) [101].

The suggested mechanism discussed by the authors may be through inhibition of TB DNA-dependent RNA polymerase (RNAP) activity through binding to the β′-subunit of the polymerase. This site is located 13–18 Å away from the binding site of RIF in this subunit. Studies indicate that the compound chelates with magnesium ions (Mg^2+^) in a similar way to that required for inhibiting the active site of HIV-1 IN [101].

**Figure 20:**
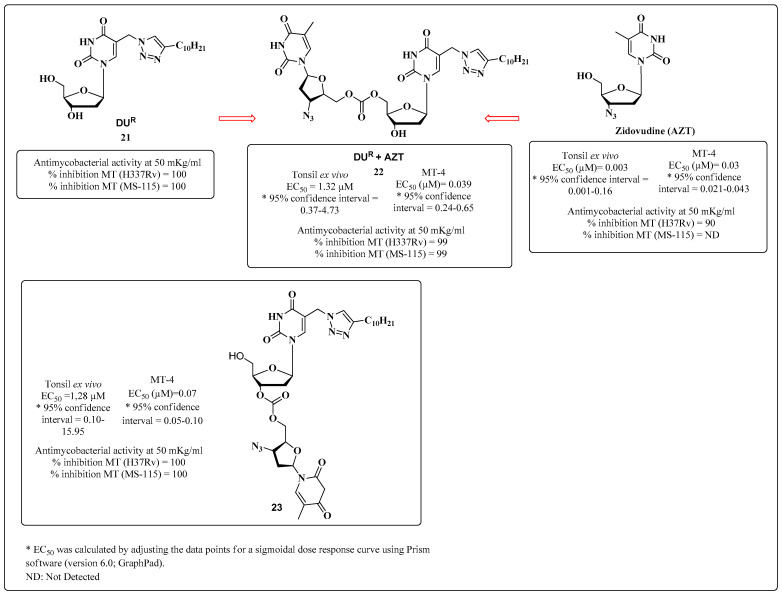
The anti-HIV and anti-MTB activities of Compounds **22** and **23** [102].

In 2017, Alexandrova and coworkers reported compounds [102] with chemical structures based on the DUR derivative **21**, which had been previously synthesized by Shmalenyuk and coworkers [103]. Alexandrova et al. reported hybridization between DUR+AZT (**22**) and between AZT+5-arylaminouracil (**23**), as well as the anti-MTB activity of the compounds [104] (Figure 20). Although all the compounds exhibited dual activity in vitro (MT-4 cells) and in ex vivo HIV-1 assays, the hybrid **23** exhibited the highest HIV-1 inhibitory activity, as well as good inhibitory activity against the MS-115 drug-resistant MTB line. In addition, **23** was more active in ex vivo assays than the hybrid **22** (Figure 18) [102].

Kumar and coworkers [105] identified piperidine as a privileged structure with properties anti-HIV and anti-MTB. Kumar et al. found that 1,4-disubstituted piperidine derivatives exhibited high anti-MTB activity [106,107,108] and moderate inhibition activity against HIV-1 [109]. The same anti-MTB property was observed for the Schiff bases of the drug INH [110,111]. The authors [105] also found that aminotriazine derivatives linked to piperidine exhibit excellent activities against wild-type HIV-1 in the nanomolar range [112].

**Figure 21:**
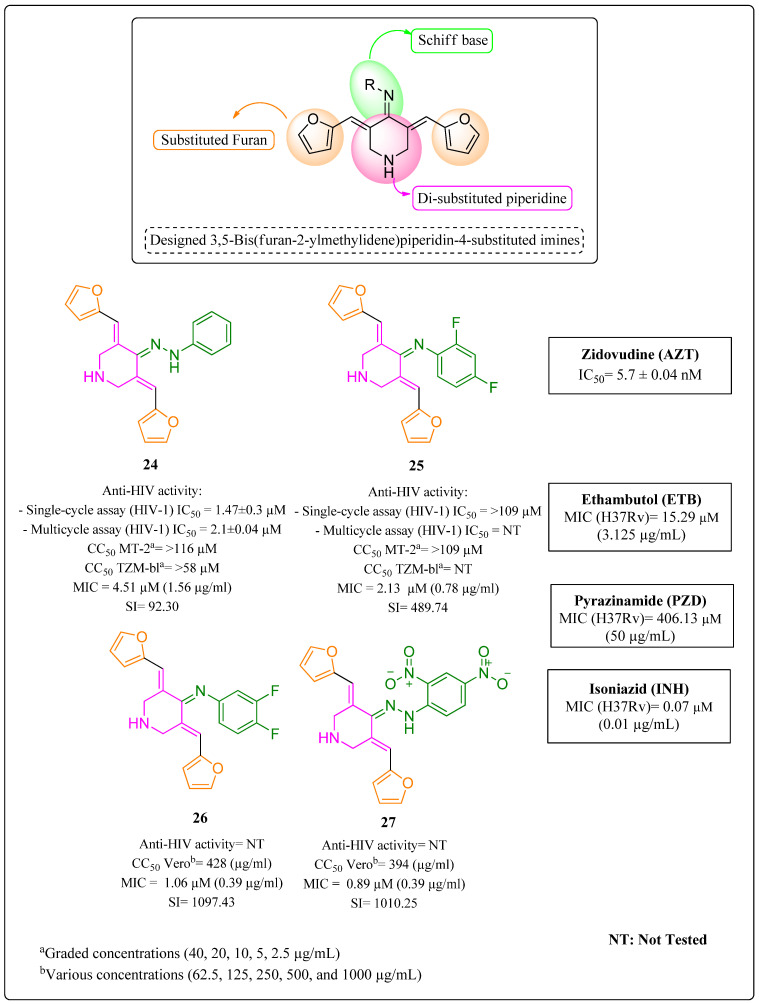
Anti-HIV and anti-MTB activities, as well as cytotoxicity studies, of 3,5-bis(furan-2-ylmethylidene)-piperidin-4-substituted imines derivatives **24**–**27** [105].

These discoveries supported the development of a series of 25 derivatives of 3,5-bis(furan-2-ylmethylidene)-piperidin-4-substituted imines. All the substances were tested for anti-MTB activity in vitro against the H37Rv strain and for anti-HIV activity in single-cycle assays and multicycle infection assays. Compounds **24** (MIC = 1.56 μg/mL), **25** (MIC = 0.78 μg/mL), **26** (MIC = 0.39 μg/mL) and **27** (MIC = 0.39 μg/mL) were found to be more potent anti-MTB agents than drugs used as standards, ETB (MIC = 3.125 μg/mL) and PZD (MIC = 50 μg/mL). In particular, the anti-MTB activity of Compounds **26** and **27** was only four times lower than that of the drug INH (MIC = 0.1 μg/mL), which was also used as a standard (Figure 21). Only Compounds **24** and **25** were analyzed for anti-HIV activity, and Compound **24** was found to be moderately active (IC_50_ = 2.1 ± 0.04 μM) in the multicycle assay compared to the standard drug AZT (IC_50_ = 5, 7 ± 0.04 nM) (Figure 21). Of the compounds studied, only Compound **24** exhibited potential as a multitarget against HIV-TB coinfection [105].

The safety of Compounds **24** and **25** was analyzed through cytotoxicity assays in MT-2 and TZM-bl cells, respectively, that were both infected with human lymphotropic virus Type 1 (HTLV-1). Vero cells were used to evaluate Compounds **26** and **27**. All the tested compounds were found to be safe (Figure 21) [105].

The authors highlighted that the performance of a specific protein inhibition assay to define the mode of action was outside the scope of the study. However, they decided to carry out molecular docking tools to predict the possible TB and HIV protein interaction. Therefore, for anti-TB activity, the enzyme applied was mycobacterial enoyl-ACP-reductase (EACP), which is involved in mycolic acid biosynthesis. For HIV analysis, RT was the choice [105]. Results of both EACP and RT confirmed 24 as the most promising multitarget agent of the series.

To design and analyze novel NNRTIs with similar activity against MTB, Chitre and colleagues [113] synthesized a series of seven novel thiazolidin-7-one derivatives and evaluated their anti-HIV and anti-MTB activities in 2019. In addition to having anti-MTB action, the thiazolidinone subunit has a broad spectrum of activities and occurs in compounds that have been synthesized and evaluated as NNRTIs against HIV-1 [114,115,116].

**Figure 22:**
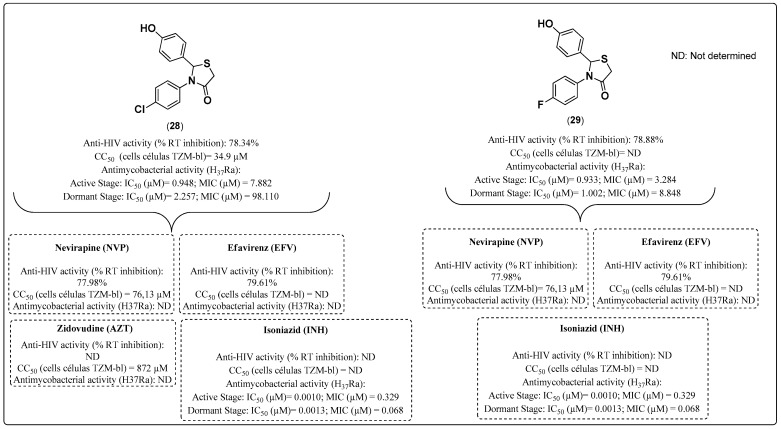
Compounds **28** and **29** and their anti-HIV and anti-MTB activities reported by Chitre and collaborators in 2009 [113].

An in vitro RT assay was performed to screen the synthesized compounds for anti-HIV-1 activity, and the measured activities were reported as percentages of inhibition at 100 μg/mL using NVP and EFV as standard drugs (at a concentration of 100 μg/mL). The screening results indicated that compounds **28** and **29** inhibited HIV-1 RT by 78.34% and 78.88%, respectively, which was comparable to that of NVP (77.98%) and EFZ (79.61%). Compounds **28** and **29** also exhibited anti-MTB potential against active and dormant MTB H37Ra species. Compound **29** exhibited considerable anti-MTB activity against the active species (IC_50_ = 0.93 μM; MIC = 3.28 μM) and the dormant species (IC_50_ = 1.00 μM; MIC = 8.84 μM). Furthermore, Compound **28** exhibited good anti-MTB action against the active species (IC_50_ = 0.94 μM; MIC = 7.88 μM) and the dormant species (IC_50_ = 2.25 μM; MIC = 98.11 μM). Compound **28** exhibited a CC_50_ of 34.9 μg/mL in a cytotoxicity assay on TZM-bl cells; however, the CC_50_ of Compound **29** was not calculated (Figure 22) [113].

From the docking simulation, it was possible to verify that these molecules have a significant affinity for HIV-1 RT and the active site of the crucial cell-wall target mycobacterial enoyl-ACP reductase (InhA) [113].

**Figure 23:**
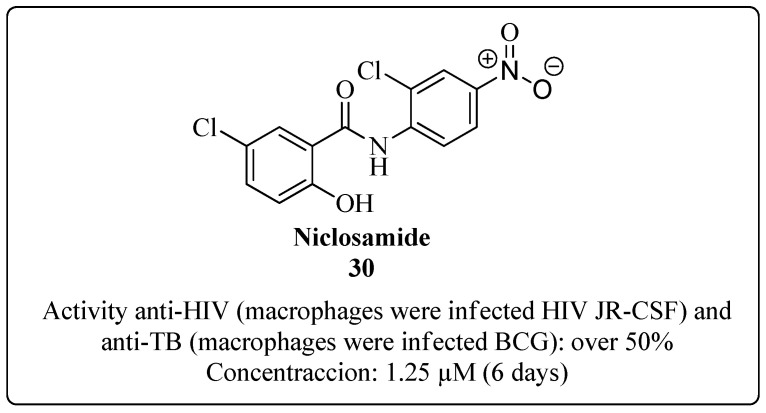
Niclosamide **30** is a potential dual anti-MTB and anti-HIV drug [74].

Niclosamide **30** (Figure 23) is a drug used to treat parasitic infections. However, there are reports that this drug has the potential to treat bacterial and viral infections, because its ability to inhibit the growth of MTB has already been demonstrated [74,117]. In a recent example of drug repositioning, Fan and collaborators verified in 2019 the dual activity of niclosamide in human macrophages coinfected with MTB and HIV-1. An in vitro coinfection system with primary cells was used. Macrophages arising from monocytes infected with HIV JR-CSF (a variant of HIV Type 1) and BCG were cultured in the presence or absence of niclosamide at a concentration of 1.25 μM. The use of niclosamide under these conditions provided a reduction of over 50% of the replication of both pathogens, showing no relevant cell death due to the toxicity of the drug. These results indicate that niclosamide has promising dual activity and can be used to treat coinfection of HIV-TB [74].

## 3. Conclusions and Future Directions

A series of problems are encountered for therapy of HIV-TB coinfection because the treatments of both diseases are based on cocktails that are difficult to adhere to. This low adhesion facilitates mutations of both pathogens, necessitating changes in the therapeutic regime by the introduction of other drugs, which in most cases present higher toxicity than the original drugs. The administration of several drugs produces many undesirable adverse effects in the coinfected individual due to the overlap of toxicities of the two concomitant therapies. RIF is a first-line drug used to treat TB that shows antiretroviral drug interactions. Thus, the best drug regimen for the coinfected patient must be carefully selected.

One alternative to improve adherence to HIV-TB therapy is the combination of drugs in FCD. However, there is still no FCD available to treat HIV-TB coinfection. This situation may result from the complexity involved in developing formulations containing more than one API.

Multitarget molecules that can act concomitantly against HIV-1 and MTB provide a very promising tool that could solve or reduce the problems of treating HIV-TB coinfection. Multitargets may act synergistically toward both pathologies, increasing adherence to therapy by providing a simplified therapeutic scheme. Moreover, multitargets would minimize the occurrence of overlapping toxicity and drug interactions, which are the leading cause of interruption of therapy.

It may be difficult to adjust the therapeutic dose of a multitarget drug because it may be sufficient to treat one pathogen and minimally combat the other pathogen. In this review, we found only one article that used a coinfected cellular model to investigate the pharmacological activity of the compounds. These factors may explain the difficulty in advancing to clinical trials of compounds planned as multitarget HIV-TB. However, therapies to reduce deaths from HIV-TB coinfection need to be urgently developed because a multitarget drug that can treat both diseases have not yet been found.

Research is being carried out to find such a multitarget drug, with promising results thus far. This report is the first review on molecules with activities against HIV and MTB that could be used within a multitarget strategy to treat HIV-TB coinfection. The antiretroviral drugs EFV, NVP, AZT, d4T and NFV and the anti-MTB drugs INH, RIF, NOR and CIP were used in different multitarget compounds. The use of niclosamide as an antiparasitic was recently demonstrated in vitro as an anti-HIV and anti-MTB agent. Niclosamide is a promising prototype for novel molecules with dual activity or can even be a candidate for clinical trials.

Additional studies are needed to evaluate the in vivo impact of this promising class. We aimed to provide an outlook from the existing data on multitarget molecules against HIV-TB coinfection that might help medicinal chemists in the future design and development of this class for in vitro and in vivo studies so that new drug candidates can be introduced to the market and into clinical use.

## Figures and Tables

**Table 1:** Recommendations for the simultaneous use of ARV and tuberculostatics [12,46].

ART and Tuberculostatic Combinations	Indication	Dosage
TDF/^a^ 3TC/DTG + tuberculostatic with RIF	First choice	TDF/3TC: 300 mg tablet/day DTG: 50 mg (2 × day)
TDF/3TC/EFV + tuberculostatic with RIF	Alternative	DFC: 300 mg + 300 mg + 400 mg/day
TDF/3TC/^b^ PI + tuberculostatic with rifabutin (RFB)	Alternative	RFB: 150 mg/day
TDF/3TC/^c^ LPV-r folded dose + tuberculostatic with RIF	Alternative	-

^a^ 3TC = Lamivudine. ^b^ PI = Protease inhibitor. ^c^ LPV-r = Lopinavir-ritonavir.

**Table 2:** Activity of (+)-calanolide A against susceptible and resistant strains to anti-MTB drugs [77].

MIC (μM) (Resistance Multiplication) ^a^					
MTB Strain	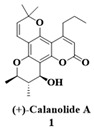	INH	RIF	SMC	ETB
H_37_Ra	43	0.45	ND	ND	19.57
H_37_Rv	21	0.22	0.019	0.42	9.78
CSU ^b^ 19	43	0.22	0.019	0.42	9.78
CSU 33	43	0.22	0.009	0.42	19.57
H37Rv-INH-R	21 (1)	>933 (>4100)	0.037 (2)	0.42 (1)	39.15 (4)
CSU 36	21 (1)	0.22 (1)	77 (4000)	0.42 (1)	9.78 (1)
CSU 38	21 (1)	0.22(1)	0.019 (1)	>220 (>512)	9.78 (1)
H_37_Rv-EMB-R	21 (1)	1.82 (8)	0.037 (2)	0.42 (1)	313 (32)

Data taken from Reference [60]. ^a^ The resistance multiplier is the ratio between the MIC value against the individual strains to the MIC value against the strain H_37_Rv. ^b^ CSU: Colorado State University (University of Colorado). ND: Not determined.

## Data Availability

Not applicable.
